# The extremely high diversity of Collembola in relict forests of Primorskii Krai of Russia

**DOI:** 10.3897/BDJ.9.e76007

**Published:** 2021-11-19

**Authors:** Nataliya Kuznetsova, Anna Bokova, Alexander Kuprin, Mikhail Potapov, Yulia Shveenkova, Natalya Ivanova

**Affiliations:** 1 Moscow State Pedagogical University, Moscow, Russia Moscow State Pedagogical University Moscow Russia; 2 Federal Scientific Center of the East Asia Terrestrial Biodiversity Far Eastern Branch of the Russian Academy of Sciences, Vladivostok, Russia Federal Scientific Center of the East Asia Terrestrial Biodiversity Far Eastern Branch of the Russian Academy of Sciences Vladivostok Russia; 3 Privolshkaya Lesostep Nature Reserve, Penza, Russia Privolshkaya Lesostep Nature Reserve Penza Russia; 4 Institute of Mathematical Problems of Biology RAS – the Branch of Keldysh Institute of Applied Mathematics of Russian Academy of Sciences, Pushchino, Russia Institute of Mathematical Problems of Biology RAS – the Branch of Keldysh Institute of Applied Mathematics of Russian Academy of Sciences Pushchino Russia

**Keywords:** sampling event, springtails, soil fauna, mesofauna, microarthropods, broad-leaf – cedar pine forests, natural reserves, multi-scale sampling design, species richness, population density, Ussuriiskii natural reserve, Sikhote-Alinskii natural reserve, Kedrovaya Pad’ natural reserve

## Abstract

**Background:**

The diversity of soil animals of relict forest ecosystems in East Asia continues to be insufficiently studied and almost not represented in international databases, including GBIF. This article is based on 7550 records of 175 species which were collected in Ussuriiskii, Sikhote-Alinskii and Kedrovaya Pad’ natural reserves of Russian Far East in 2016–2017. A multi-scale sampling design allowed us to estimate population densities and local species richness of Collembola at areas of different sizes. The work continues the digitization of the collections of the Moscow Pedagogical State University (MPSU) and their publication through GBIF.org, which began in 2019. This article is based on original data including 2377 specimens of springtails from eight forests and 648 soil cores.

**New information:**

Within the framework of modern taxonomy, this work represents the first publication of lists of Collembola species of forests of Primorsky Krai. The work focused on the relict protected cedar-deciduous forests. Nine species new to science were described and data on the fauna of the region were significantly revised. Considerable contribution was made to the biogeography of Collembola of East Asia. The design of the sampling allowed us to draw conclusions about the species saturation of springtails at various spatial scales within the habitat: from a few square cm to 100 sq. m. Number of species reached record high values reflecting the benchmark state of Collembola communities of undisturbed old temperate forest ecosystems.

## Introduction

Collembola, or springtails, is one of the most abundant and diverse groups of soil microarthropods that play an important role in the processes of destruction of organic residues (Petersen and Luxton 1982, Hopkin 1997). We aimed to estimate the upper limit of the local species diversity of Collembola in a region known for its high species richness of various groups of organisms (Latham and Ricklefs 1993). Part of this region is located in the south of the Russian Far East, where broad-leaf – cedar pine forest ecosystems are distributed. The highest diversity of insects in the temperate zone is described for these forests (Chernov et al. 2011). UNESCO included this area in the World Heritage List (Central Sikhote-Alin) noting that “the combination of glacial history, climate and relief has allowed the development of the richest and most unusual temperate forests in the world”.

Regular research of Collembola in the south of the Primorskii Krai of Russia began about half a century ago showing a great originality of the fauna (e.g. Kutyreva 1979, Kutyreva 1984, Martynova 1988). During the last decades, new approaches to taxonomy have led to the taxonomic revision of many genera of springtails (Pomorski and Sveenkova 2006, Deharveng et al. 2011, Jie et al. 2011, Jordana et al. 2011, Huang and Potapov 2012, Smolis et al. 2012, Smolis and Deharveng 2015). However, these works did not focus on the issues of local species richness.

We collected springtails in the region in the years 2016–2017. The data from 2016 was published (Kuznetsova et al. 2019). However, the species list was limited to abundant species only. The present study includes the full species list, based on samples from 2016 and 2017 taking into account the recently described new species for science. A specific multi-scale sampling design allowed us to estimate a local species richness of Collembola at areas of different sizes.

## General description

### Purpose

The purpose of the paper is to present information on species composition and abundances of Collembola in relict broad-leaf – cedar pine forest ecosystems of the Russian Far East. Diversity and аbundance are presented in the most detailed form of sampling-evidence.

## Sampling methods

### Study extent

The dataset (Kuznetsova et al. 2021) provides information on the number of individuals of springtail species collected in broad-leaf – cedar pine forests of three natural reserves in July 2016 and August 2017 (7550 occurrences). Three forests were examined in Ussuriiskii natural reserve, 2 – in Kedrovaya Pad’, 2 – in Sikhote-Alinskii natural reserve and 1 – in Chuguevsky District. The sampling plots “Fauri” and “Grabovaya” were in mountain forest, plots “Turova” and “Kedrovaya” were on a slope, plots “Pikhtovaya”, “Kema”, “Chuguev” and "Anikin" were in the river valleys. The material includes about 24 thousands individuals of 175 species from eight sampling series and 648 soil cores. They were collected by Natalia Kuznetsova, Mikhail Potapov, Anna Geraskina, Alexander Kuprin, Anastasia Korotkevich and were identified by Natalia Kuznetsova, Mikhail Potapov, Babenko Anatoly, Shveenkova Yulia.

### Sampling description

The sampling was based on a multi-scale design to study the structure of biodiversity at different spatial scales (Lande 1996, Azovsky et al. 2000). A fractal arrangement of cores allows us to reduce sample effort because the same core is used for the analysis at different scales (Marsh and Ewers 2012). A few cases of applying the approach in soil zoology include testate amoebae (Tsyganov et al. 2014), oribatid mites (Bolger et al. 2014) and springtails (Kuznetsova and Saraeva 2018). We used the small size of the corer (8 cm^2^ in section) to implement special attention on the diversity and spatial structure of the population at the micro level. Soil was investigated down to 20 cm. A total of 81 cores were taken in each sampling plot. Cores were placed in the corners of different-scale equilateral triangles inscribed in squares with sides 10 cm, 25 cm, 1 m and 10 m. The different-scale triangles were designed following the principles of fractal geometry. The sample design is described in detail by Saraeva et al. (2015).

**Figure 1.**
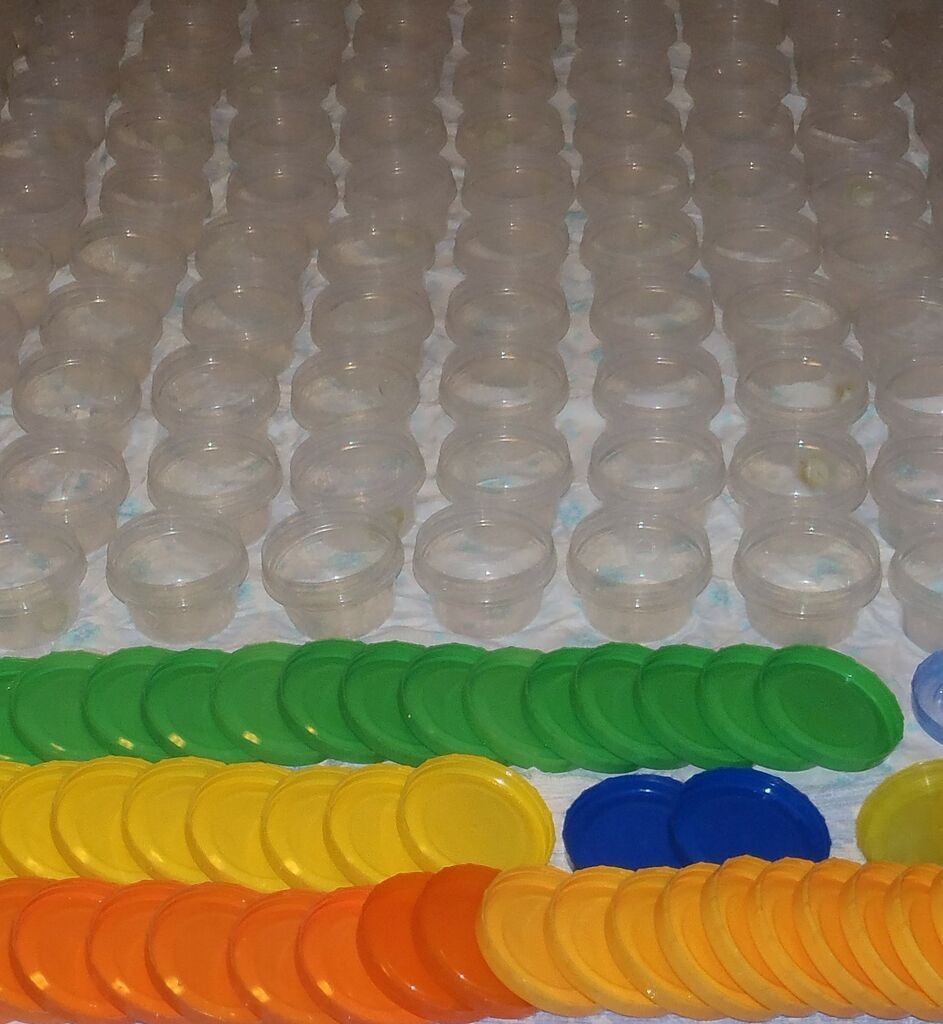
Plastic containers

**Figure 2.**
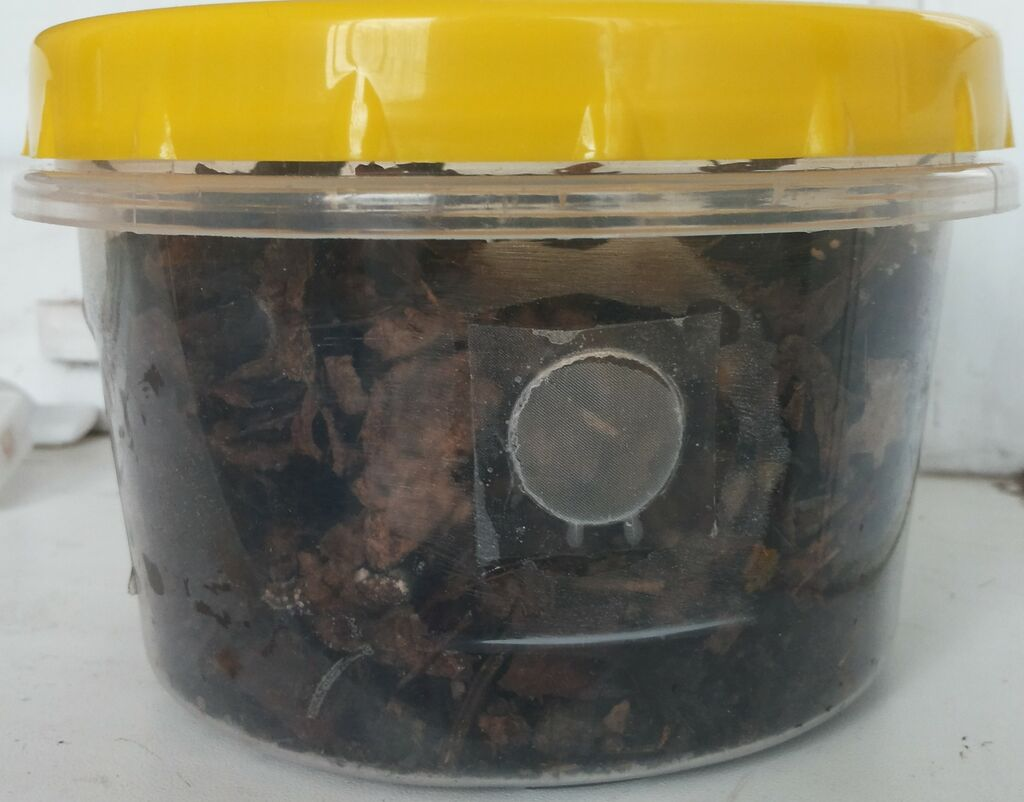
One plastic container with a core

**Extraction of Collembola from cores**: Plastic containers were used for storage and transportation of individual cores (Fig. 1). Each core was placed in a 0.3 litre plastic container with a vent hole covered with a gas cloth (Fig. 2). The containers were transported to MPGU (Moscow). Extraction of Collembola was performed in the laboratory using Tullgren's funnels at approximately 25^o^C. Extraction into 70% alcohol was continued for 4-5 days until the cores were completely dry.

**Laboratory processing**: All the specimens were mounted on slides in Phoera liquid according to a standard procedure (Ghilarov 1975, Potapov and Kuznetsova 2011).

**Sampling plots**: Short descriptions of some sampling plots (“Turova”, “Grabovaya”, “Kedrovaya”, “Pikhtovaya”) were published (Kuznetsova et al. 2019). The descriptions of the other plots are given for the first time. All the plots were located on brown soil.

**Figure 3.**
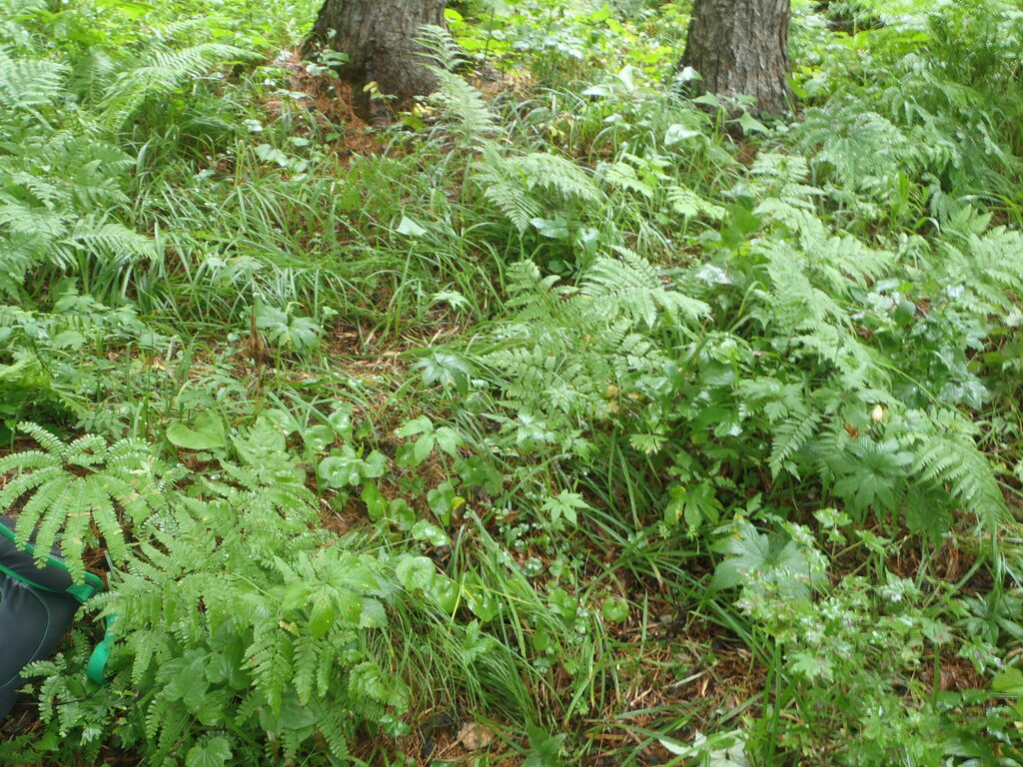
Above-soil cover in broad-leaf – cedar pine forest in Ussuriiskii natural reserve (plot “Turova”), photo by N. Kuznetsova.

**Figure 4.**
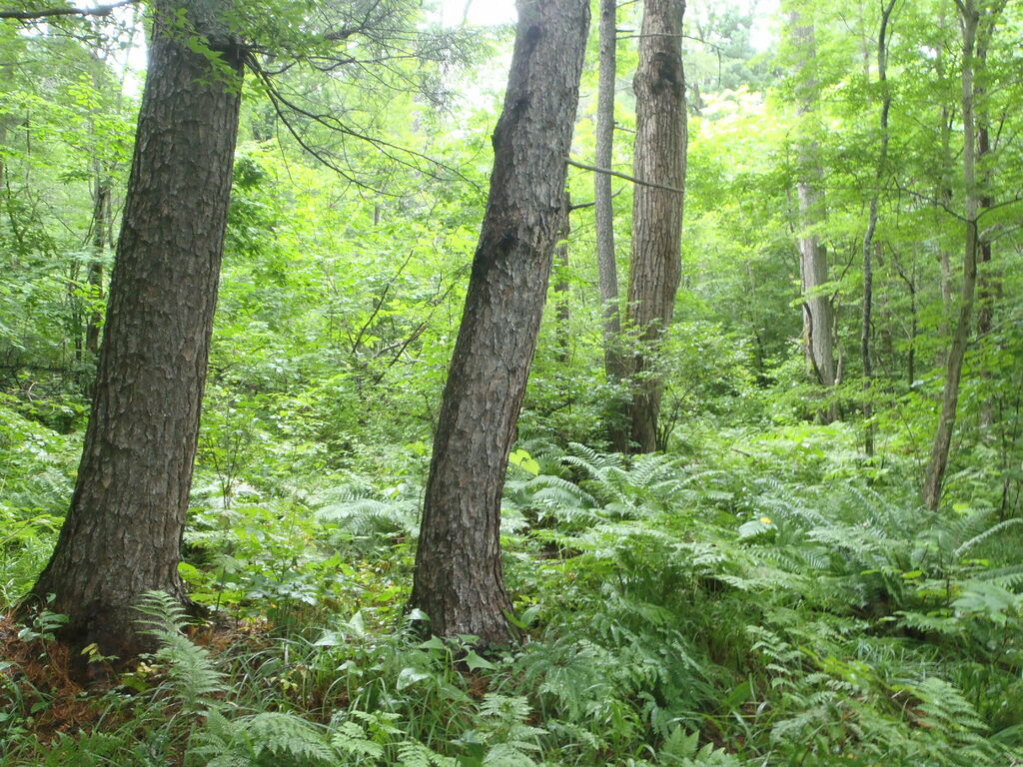
Above-soil cover in broad-leaf – cedar pine forest in Ussuriiskii natural reserve (plot “Turova”), photo by N. Kuznetsova.

The sampling plot “Turova” (Figs 3, 4) is in the Ussuriiskii natural reserve; it is a cedar pine–deciduous forest on a flat slope (*Pinuskoraiensis* Siebold & Zucc., *Acermandshuricum* Maxim., *Quercusmongolica* Fisch. etc.); in the undergrowth *Loniceramaackii* (Rupr.) Maxim., *Eleutherococcussenticosus* (Rupr. & Maxim.) Maxim. etc.; in the above-soil cover *Oxalisacetosella* L., *Carex* L., *Adiantumpedatum* L. etc. The dead cover was ~10 cm.

**Figure 5.**
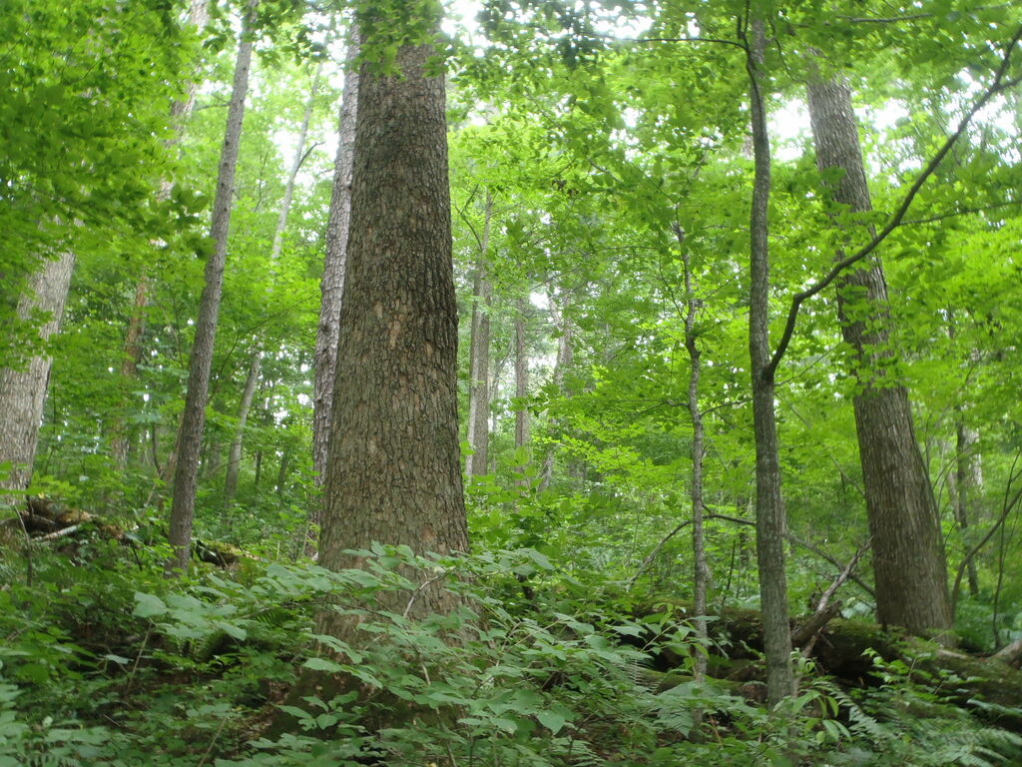
Mountain fir–hornbeam forest in Ussuriiskii natural reserve (plot “Grabovaya”), photo by N. Kuznetsova.

The sampling plot “Grabovaya” (Fig. 5) is in the Ussuriiskii natural reserve; it is on Mount Grabovaya, in fir–hornbeam forest (*Abiesholophylla* Maxim., *Carpinuscordata* Blume, *Pinuskoraiensis*, *Betulacostata* Trautv. etc.); in the undergrowth are *Caprinuscordata*, *Acertegmentosum* Maxim., *Acerbarbinerve* Maxim. ex Miq., *Eleutherococcussenticosus* etc.; in the above-soil cover are *Oxalisacetosella*, *Leptorumohraamurensis* (Milde) Tzvelev etc. The dead cover is > 80% and the thickness of the litter is ~ 4 cm.

The sampling plot “Kedrovaya” is in the Kedrovaya Pad’ natural reserve; it is a cedar pine–fir broadleaved forest on a slope (*Pinuskoraiensis*, *Abiesholophylla*, *Tiliamandshurica* Rupr. & Maxim., *Carpinuscordata* etc.); in the undergrowth are five maple species, including *Acertegmentosum* and *Acerbarbinerve*; in the above-soil cover are *Leptorumohraamurensis*, *Dryopteriscrassirhizoma* Nakai, *Maianthemumdilatatum* (Alph.Wood) A.Nelson & J.F.Macbr., *Oxalisacetosella* etc. The dead cover ~ 50% and the litter thickness is 4 cm.

The sampling plot “Pikhtovaya” is in the Kedrovaya Pad’ natural reserve; it is a valley fir and deciduous forest (*Abiesholophylla*, *Juglansmandshurica* Maxim., *Pinuskoraiensis* etc.); in the undergrowth are *Juglansmandshurica*, *Carpinuscordata*, *Acermono* Maxim., *Acertegmentosum*, *Acermandshuricum* etc.; in the above-soil cover are *Leptorumohraamurensis*, *Dryopteriscrassirhizoma* etc. The dead cover is ~ 50% and the thickness of the litter is 3–4 cm.

**Figure 6.**
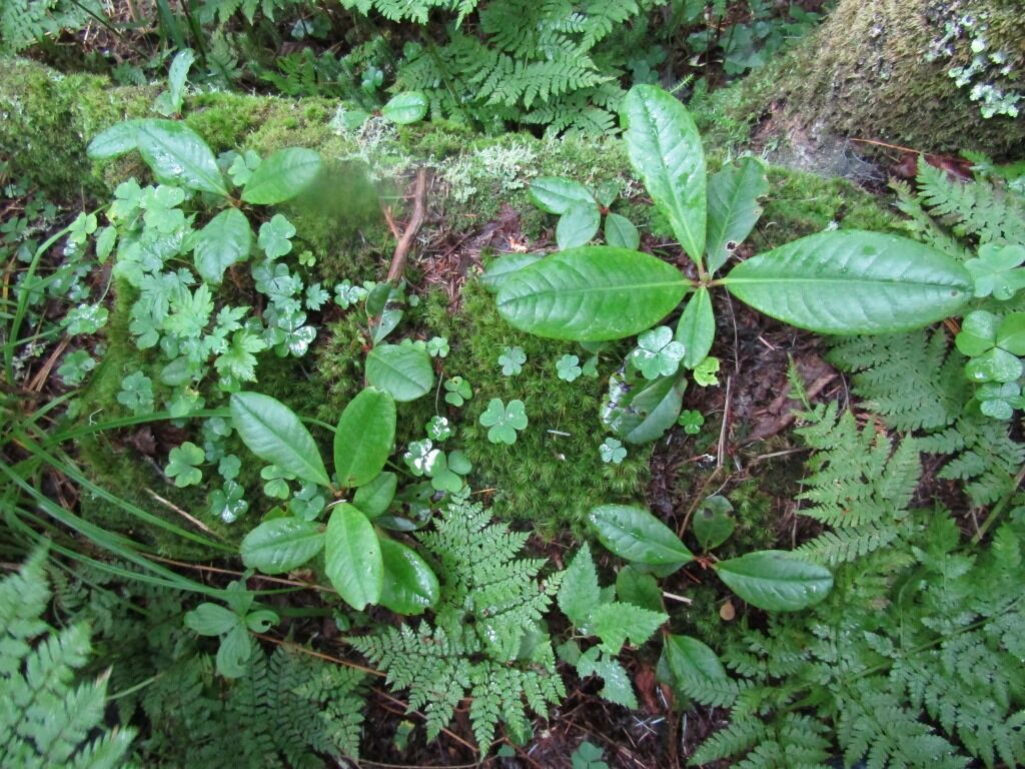
Above-soil cover in mountain coniferous forest with *Rhododendromfauriei* (plot “Fauri”) in Sikhote-Alinskii natural reserve, photo by A. Geras'kina.

**Figure 7.**
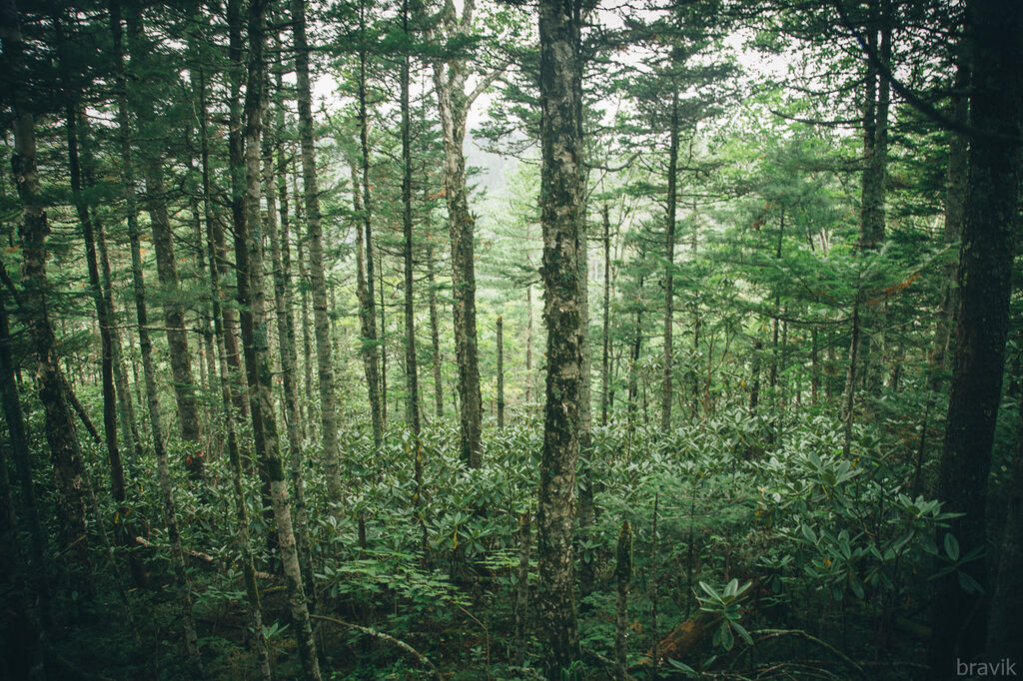
Mountain coniferous forest with *Rhododendromfauriei* (plot “Fauri”) in Sikhote-Alinskii natural reserve, photo by R. Naumenko.

The sampling plot “Fauri” (Figs 6, 7) is in the Sikhote-Alinskii natural reserve, Kabani station, at 932 m alt.; it is a coniferous wood (*Abiesnephrolepis* (Trautv. ex Maxim.) Maxim., *Betulaplatyphylla* Sukaczev, *Pinuskoraiensis)*; in the undergrowth are *Rhododendromfauriei* Franch., *Acerukurunduense* Trautv. & C.A.Mey., *Piceajezoensis* (Siebold & Zucc.) Carrière; in the above-soil cover are *Leptorumohraamurensis*, *Oxalisacetosella*, *Maianthemumbifolium* (L.) F.W.Schmidt etc. The dead cover is 50–100% and the thickness of the litter is 3–7 cm.

The sampling plot “Kema” is nearby the Sikhote-Alinskii natural reserve, in the valley of Brusnichnaya River (tributory of the Kema); it is mixed forest (*Pinuskoraiensis*, *Populusmaximowiczii* A.Henry, *Piceajezoensis*, *Ulmusglabra* Huds., *Abiesnephrolepis*); in the undergrowth are *Acermono*, *Acerbarbinerve*, *Acertegmentosum*, *Philadelphustenuifolius* Rupr. & Maxim., *Eleutherococcussenticosus* etc.; in the above-soil cover are *Leptorumohraamurensis*, *Oxalisacetosella*, *Maianthemumbifolium*, *Carexsiderosticta* Hance, *Cardamineleucantha* (Tausch) O.E.Schulz, *Cacaliahastata* L. etc. The dead cover is 5–65% and the thickness of the litter is 7–10 cm.

**Figure 8.**
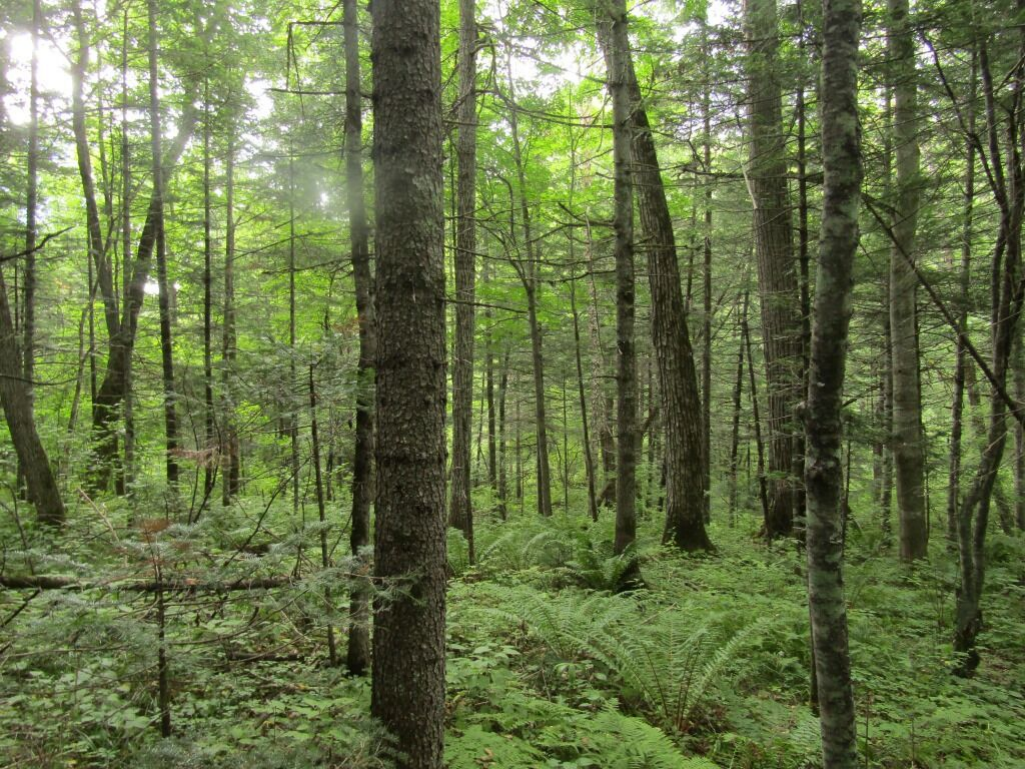
Valley mixed forest (plot “Chuguev”), photo by A. Geras'kina.

**Figure 9.**
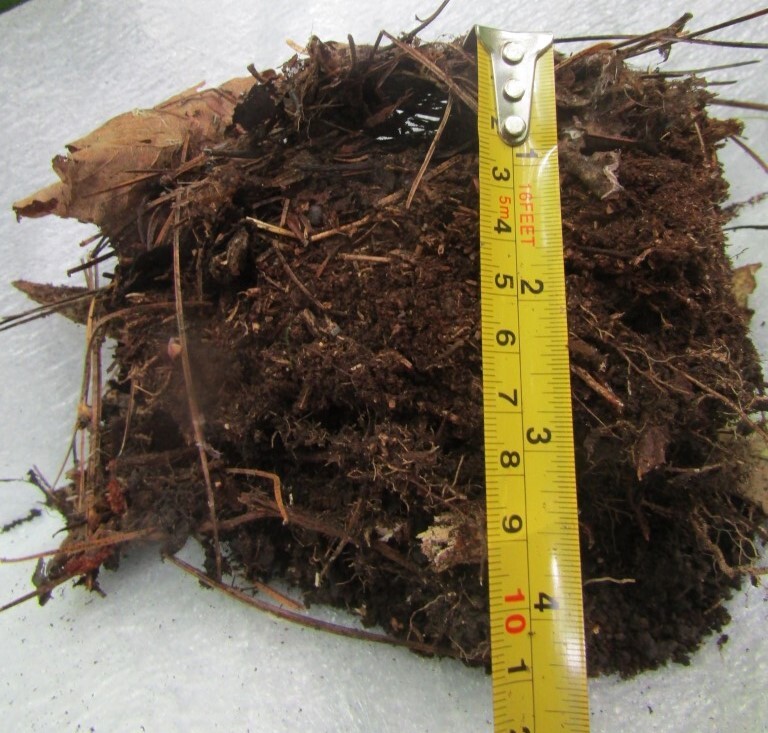
Litter in plot “Chuguev”, photo by A. Geras'kina.

The sampling plot “Chuguev” (Figs 8, 9) is in the Chuguevski District near Verkneussuriyski Station of the Federal Scientific Center of the East Asia Terrestrial Biodiversity; it is a valley mixed forest (*Abiesnephrolepis*, *Populusmaximowiczii*, *Fraxinusmandshurica* Rupr., *Betulacostata*, *Pinuskoraiensis* etc.); in the above-soil cover are *Abiesnephrolepis, Acermono, Acertegmentosum, Loniceramaackii, Philadelphustenuifolius* etc.; in the above-soil cover are *Oxalisacetosella, Leptorumohraamurensis*, *Carexcampylorhina* V.I.Krecz., *Cardamineleucantha*, *Athyriumrubripes* (Kom.) Kom. etc.). The dead cover is 20–85% and the thickness of the litter is 6–9 cm.

The sampling plot “Anikin” is in the Ussuriyskii natural reserve, Suvorovskoye forest district, Anikinsky station, valley of Anikin River; it is valley broadleaf forest (*Juglansmandshurica*, Populusmaximowiczii, *Fraxinusmandshurica*) with *Pinuskoraiensis*; in the above-soil cover are *Carex* L., *Leptorumohraamurensis* etc. The dead cover is 50–90% and the thickness of the litter is 3–4 cm.

### Quality control

We used both modern taxonomic papers and keys (Martynova 1988, Babenko et al. 1994, Potapov 2001) for the taxonomic determination of Collembola. The material was checked by leading experts in taxonomy of Collembola. Scientific names were checked using the GBIF species matching tool.

### Step description

Data on species were digitised, standardised according to the Darwin Core (Wieczorek et al. 2012), the quality of the data was checked and errors were corrected and then published through GBIF.org (Kuznetsova et al. 2021).

## Geographic coverage

### Description

**Figure 10.**
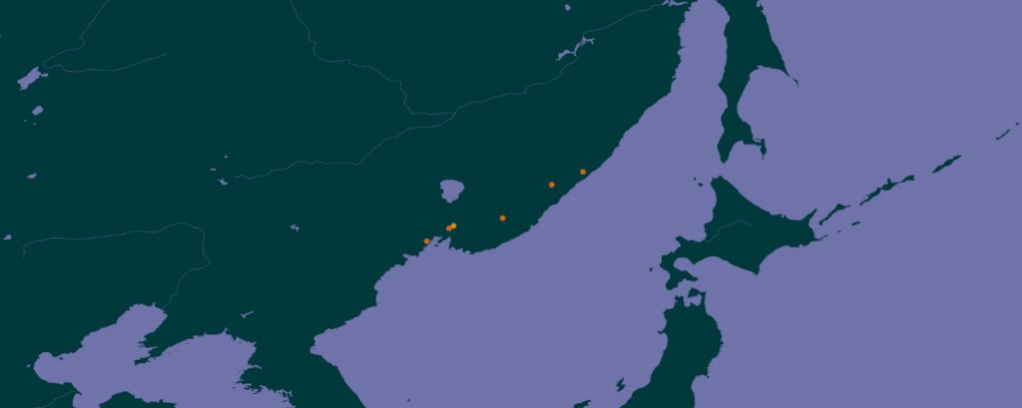
Geographic coverage. Study areas in the Primorskii Kraii (Kuznetsova et al. 2021, doi.org/10.15468/dyadwn).

Primorskii Krai of the Russian Far East (Fig. 10).

### Coordinates

43.115 and 45.648 Latitude; 131.487 and 137.01 Longitude.

## Taxonomic coverage

### Description

So far, the taxonomical knowledge of different families and genera of Collembola is highly irregular in the area under study. Our identification of particular groups of Collembola, therefore, considerably depended on taxa. The species of Neelidae, Symphypleona, Lepidocyrtinae and Entomobryini were identified, based on the appearance (body size, colour pattern, length of limbs and other easily recognisable features), other taxa - on modern taxonomy, family Tomoceridae - on traditional characters. Families Hypogastruridae, Onychiuridae and Isotomidae were identified down mostly to species level, while the genera *Isotoma* and *Desoria* still are less certain and were differentiated as morpho-species (sp. 1, sp. 2 etc). Family Odontellidae is less understood in the area and so it was mostly represented by the "sp." in the list. Some species were described as new to science in the material: *Anurida* - 6 spp. n. (Babenko et al. 2019), *Oligaphorura* – 2 spp. n. (Xin et al. 2019); *Pseudachorutes* – 1 sp. n. and re-description of three species (Babenko et al. 2021).

### Taxa included

**Table:** 

Rank	Scientific Name	
phylum	Arthropoda	
class	Collembola	

## Temporal coverage

**Data range:** 2016-7-23 – 2016-7-29; 2017-8-06 – 2017-8-13.

## Usage licence

### Usage licence

Other

### IP rights notes

Creative Commons Attribution (CC-BY) 4.0 License

## Data resources

### Data package title

Collembola of the relict forests of the Russian Far East.

### Resource link

https://www.gbif.org/dataset/321e6294-7e96-44c2-ac5d-6b009ef17618

### Number of data sets

1

### Data set 1.

#### Data set name

Collembola of the relict forests of the Russian Far East.

#### Data format

Darwin Core Archive

#### Number of columns

37

#### Character set

UTF-8

**Data set 1.** 

Column label	Column description
eventID	An identifier for the event https://dwc.tdwg.org/terms/#dwc:eventID
samplingProtocol	Sampling protocol (Tullgren funnels) https://dwc.tdwg.org/terms/#dwc:samplingProtocol See details in the Sampling methods section.
sampleSizeValue	Size of the sampling core (8 cm^2^). https://dwc.tdwg.org/terms/#dwc:sampleSizeValue See details in the Sampling methods section.
sampleSizeUnit	The unit of measurement of the size sampling core (cm^2^) https://dwc.tdwg.org/terms/#dwc:sampleSizeUnit See details in the Sampling methods section.
decimalLatitude	The geographic latitude in decimal degrees of the geographic centre of the data sampling place. https://dwc.tdwg.org/terms/#dwc:decimalLatitude
decimalLongitude	The geographic longitude in decimal degrees of the geographic centre of the data sampling place. https://dwc.tdwg.org/terms/#dwc:decimalLongitude
geodeticDatum	Spatial reference system (SRS) upon which the geographic coordinates given in decimalLatitude and decimalLongitude are based. https://dwc.tdwg.org/terms/#dwc:geodeticDatum
coordinateUncertaintyInMetres	The maximum uncertainty distance in metres. https://dwc.tdwg.org/terms/#dwc:coordinateUncertaintyInMeters
coordinatePrecision	The fraction of a degree corresponding to the number of significant digits in the source coordinates. https://dwc.tdwg.org/terms/#dwc:coordinatePrecision
country	Country name (Russian Federation). https://dwc.tdwg.org/terms/#dwc:country
countryCode	The standard code for the Russian Federation according to ISO 3166-1-alpha-2 (RU). https://dwc.tdwg.org/terms/#dwc:countryCode
stateProvince	Region name. The first level administrative division. https://dwc.tdwg.org/terms/#dwc:stateProvince
locality	The specific description of the place. https://dwc.tdwg.org/terms/#dwc:locality
locationID	An identifier for the set of location information https://dwc.tdwg.org/terms/#dwc:locationID We used this term to link cores (events) in the sampling plot.
habitat	A description of the habitat in which the Event occurred https://dwc.tdwg.org/terms/#dwciri:habitat We indicated habitat type as a landscape position (valley or mountain) and tree species dominates in the canopy.
verbatimEventDate	The verbatim original date of the Event occurred. https://dwc.tdwg.org/terms/#dwc:verbatimEventDate
year	The four-digit year of the Event occurred. https://dwc.tdwg.org/terms/#dwc:year
month	The integer month of the Event occurred. https://dwc.tdwg.org/terms/#dwc:month
day	The integer day of the month of the Event occurred. https://dwc.tdwg.org/terms/#dwc:day
eventDate	Field data collection date (YYYY-MM-DD). https://dwc.tdwg.org/terms/#dwc:eventDate
institutionCode	The acronym of the Institute. https://dwc.tdwg.org/terms/#dwc:institutionCode
institutionID	An identifier for the institution having custody of the object(s) or information referred to in the record. https://dwc.tdwg.org/terms/#dwc:institutionID
basisOfRecord	Basis of the record (PreservedSpecimen). https://dwc.tdwg.org/terms/#dwc:basisOfRecord
occurrenceID	An identifier for the record. https://dwc.tdwg.org/terms/#dwc:occurrenceID
identificationRemarks	Original identification. The dwc: verbatimIdentification was not used because it is currently not supported on the IPT. https://dwc.tdwg.org/terms/#dwc:identificationRemarks
scientificName	Scientific name. https://dwc.tdwg.org/terms/#dwc:scientificName
identificationQualifier	A brief phrase or a standard term ("cf.", "aff.") to express the determiner's doubts about the Identification. https://dwc.tdwg.org/terms/#dwc:identificationQualifier
taxonRank	The taxonomic rank. https://dwc.tdwg.org/terms/#dwc:taxonRank
kingdom	The full scientific name of the kingdom (Animalia). https://dwc.tdwg.org/terms/#dwc:kingdom
phylum	The full scientific name of the phylum. https://dwc.tdwg.org/terms/#dwc:phylum
class	The full scientific name of the class. https://dwc.tdwg.org/terms/#dwc:class
identifiedBy	List of persons, who identified collected Collembola. https://dwc.tdwg.org/terms/#dwc:identifiedBy
identificationReferences	DOI of refererences used in the identification. Used for taxa, which did not match the GBIF Backbone. https://dwc.tdwg.org/terms/#dwc:identificationReferences
lifeStage	The life stage of individuals. Here it is used for juvenile individuals indicated. https://dwc.tdwg.org/terms/#dwc:lifeStag
individualCount	The number of individuals represented in the core. https://dwc.tdwg.org/terms/#dwc:individualCount
occurrenceStatus	A statement about the presence or absence of a Taxon at a Location. https://dwc.tdwg.org/terms/#dwc:occurrenceStatus
language	A language of the resource (EN). https://dwc.tdwg.org/terms/#dc:language

## Additional information

**Figure 11.**
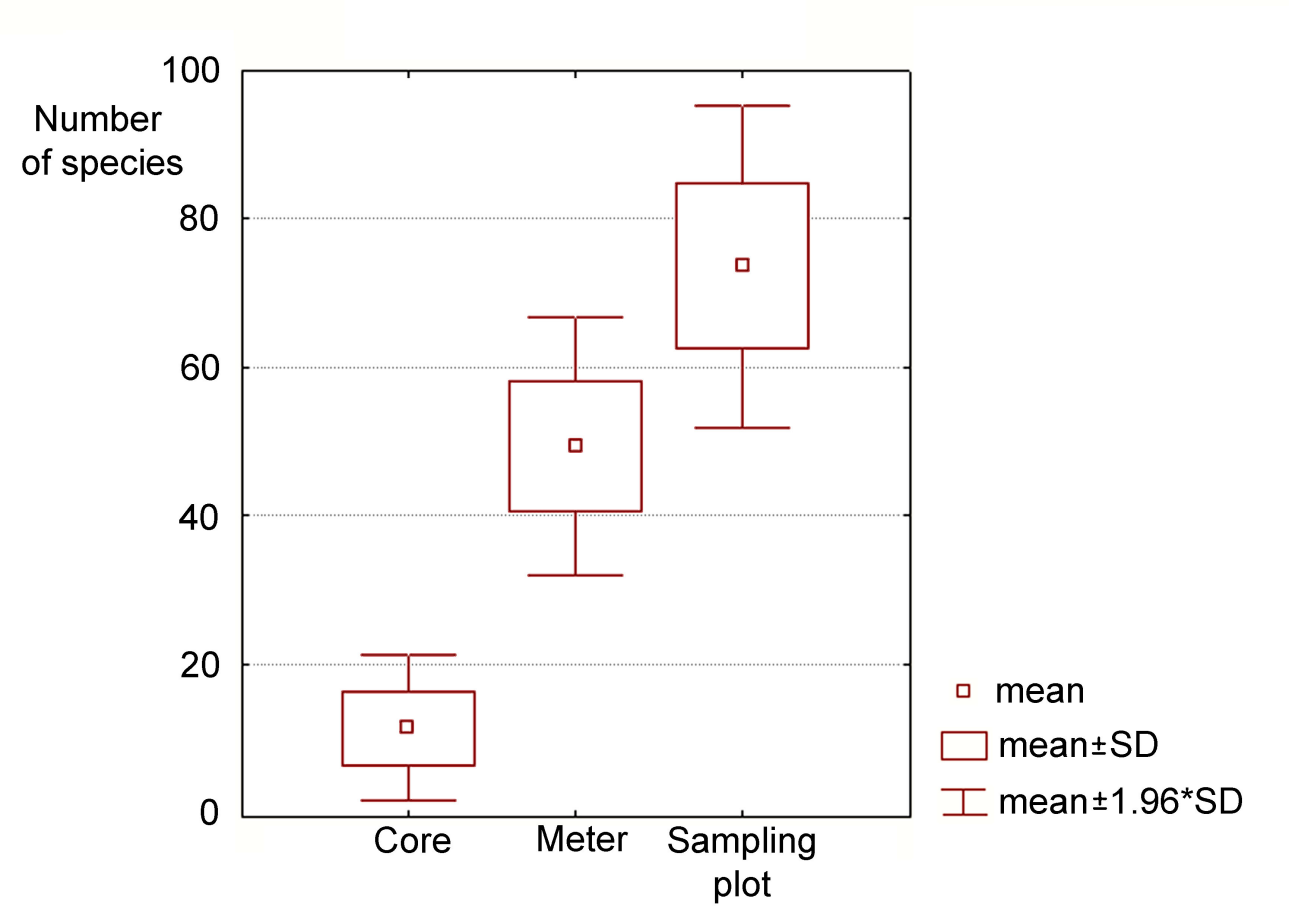
Number of Collembola species in a core, on one square metre, in one sampling plot.

In total, in the relict forests of the Far East, we found the highest diversity of Сollembola that has ever been observed in the ecosystems of the temperate zone and possibly the world: up to 90 species per area 10 x 10 m (sampling plot). The species saturation reaches 30 species on an area of 8 square centimetres (one core) and can exceed 60 species on 1 square metre (Fig. 11).

## References

[B7468027] Azovsky A. I., Chertoprood M. V., Kucheruk N. V., Rybnikov P. V., Sapozhnikov F. V. (2000). Fractal properties of spatial distribution of intertidal benthic communities. Marine Biology.

[B7468037] Babenko AB, Potapov MB, Stebaeva SK, Chernova NM (1994). Keys of springtails of the fauna of Russia and adjacent countries: family Hypogastruridae..

[B7468127] Babenko ANATOLY, Kuznetsova NATALIA, Nakamori TAIZO, Shveenkova YULIA (2021). A review of *Pseudachorutes* Tullberg, 1871 (Collembola, Neanuridae) from the East Asia, with description of six new species. Zootaxa.

[B7468099] Babenko A. B., Kuznetsova N. A., Shveenkova Yu. B. (2019). New species of the genus *Anurida* (Collembola, Neanuridae) from the Far East of Russia. Zoologicheskii Zhurnal.

[B7468145] Bolger Thomas, Arroyo Julio, Kenny Joan, Caplice Martina (2014). Hierarchical analysis of mite community structures in Irish forests—a study of the relative contribution of location, forest type and microhabitat. Applied Soil Ecology.

[B7468374] Chernov Yu. I., Lelei A. S., Storozhenko S. Yu., Lelei A. (2011). Identification guide to the Russian Far East insects. Additional volume. Analysis of the fauna and the general index of names.

[B7468479] Deharveng Louis, Bedos Anne, Weiner Wanda (2011). Two new species of the genus *Leenurina* Najt & Weiner, 1992 (Collembola, Neanuridae, Caputanurininae) from Primorskii Krai (Russia). ZooKeys.

[B7468191] Ghilarov MS, Ghilarov MS (1975). Methods of soil zoological studies.

[B7468213] Hopkin S. P. (1997). Biology of the springtails (Insecta: Collembola).

[B7468506] Huang CHENG-WANG, Potapov MIKHAIL (2012). Taxonomy of the *Proisotoma* complex. IV. Notes on chaetotaxy of femur and description of new species of *Scutisotoma* and *Weberacantha* from Asia. Zootaxa.

[B7468488] Jie DING, Potapov MIKHAIL, Sokolova ELENA (2011). Further study on the labial palp in the Isotomidae (Collembola) with reference to the genus *Heteroisotoma* Stach. Zootaxa.

[B7468497] Jordana R., Potapov M., Baquero E. (2011). New species of *Entomobryini* from Russia and Armenia (Collembola, Entomobryomorpha). Soil Organisms.

[B7468436] Kutyreva L. T.,, Dolin V. (1979). Fauna and ecology of invertebrates.

[B7468449] Kutyreva L. T., Ghilarov M. (1984). Fauna and ecology of Collembola.

[B7468204] Kuznetsova N. A., Saraeva A. K. (2018). Beta-diversity partitioning approach in soil zoology: A case of Collembola in pine forests. Geoderma.

[B7468221] Kuznetsova N. A., Bokova A. I., Saraeva A. K., Shveenkova Yu. B. (2019). Communities of Collembola in the forests of Southern Primorye as a benchmark of high diversity and organization complexity. Biology Bulletin.

[B7466754] Kuznetsova N A, Potapov M B, Shveenkova Y B, Ivanova N (2021). Collembola of the relict forests of the Russian Far East. Sampling event dataset..

[B7468239] Lande Russell (1996). Statistics and partitioning of species diversity, and similarity among multiple communities. Oikos.

[B7469790] Latham R. E., Ricklefs R. E. (1993). Global patterns of tree species richness in moist forests: energy-diversity theory does not account for variation in species richness. Oikos.

[B7468248] Marsh Charles J., Ewers Robert M. (2012). A fractal-based sampling design for ecological surveys quantifying β-diversity. Methods in Ecology and Evolution.

[B7469581] Martynova EF (1988). The order Collembola - springtails. In Keys of insects of the Far East of the USSR. V. I. Primary-winged, ancient-winged, with incomplete metamorphosis.

[B7468257] Petersen Henning, Luxton Malcolm (1982). A comparative analysis of soil fauna populations and their role in decomposition processes. Oikos.

[B7468470] Pomorski R. J., Sveenkova Y. B. (2006). New genus with three new species of *Thalassaphorurini* (Collembola: Onychiuridae) from Russian Far East. Insect Systematics & Evolution.

[B7468266] Potapov M (2001). Synopses on Palaearctic Collembola. V.3. Isotomidae..

[B7468274] Potapov MB, Kuznetsova NA (2011). Methods of research of microarthropod communities: a manual for students and postgraduates.

[B7468282] Saraeva A. K., Potapov M. B., Kuznetsova N. A. (2015). Different-scale distribution of Collembola in homogenous ground vegetation: sphagnum moss. Entomological Review.

[B7468515] Smolis A., Deharveng L., Bedos A. (2012). Description of two new species of the genus *Micranurida* Borner, 1901 (Collembola: Neanuridae, Pseudachorutinae) from Russia, with notes on the genus Lanzhotia Rusek, 1985. Entomologica Fennica.

[B7468524] Smolis ADRIAN, Deharveng LOUIS (2015). Diversity of *Paranura* Axelson, 1902 (Collembola: Neanuridae: Neanurinae) in Pacific region of Russia and United States. Zootaxa.

[B7468304] Tsyganov A. N., Komarov A. A., Mitchell E. A.D., Shimano S., Smirnova O. V., Aleynikov A. A., Mazei Y. A. (2014). Additive partitioning of testate amoeba species diversity across habitat hierarchy within the pristine southern taiga landscape (Pechora-Ilych Biosphere Reserve, Russia). European Journal of Protistology.

[B7468291] Wieczorek John, Bloom David, Guralnick Robert, Blum Stan, Döring Markus, Giovanni Renato, Robertson Tim, Vieglais David (2012). Darwin Core: an evolving community-developed biodiversity data standard. PLOS One.

[B7468230] Xin S., Shveenkova YU. B., Xie ZHIJING, Babenko A. B. (2019). New *Oligaphorura* species (Collembola: Onychiuridae) from the forests of East Asia. Zootaxa.

